# Kinetic and structural characterization of carboxyspermidine dehydrogenase of polyamine biosynthesis

**DOI:** 10.1016/j.jbc.2023.105033

**Published:** 2023-07-10

**Authors:** Danielle F. Lee, Nicole Atencio, Shade Bouchey, Madeline R. Shoemaker, Joshua S. Dodd, Meredith Satre, Kenneth A. Miller, Jeffrey S. McFarlane

**Affiliations:** Department of Chemistry and Biochemistry, Fort Lewis College, Durango, Colorado, USA

**Keywords:** polyamine, spermidine, norspermidine, carboxyspermidine, dehydrogenase, aspartate semialdehyde, putrescine, gut microbiome

## Abstract

Polyamines are positively charged alkylamines ubiquitous among eukaryotes, prokaryotes, and archaea. Humans obtain polyamines through dietary intake, metabolic production, or uptake of polyamines made by gut microbes. The polyamine biosynthetic pathway used by most gut microbes differs from that used by human cells. This alternative pathway employs carboxyspermidine dehydrogenase (CASDH), an enzyme with limited characterization. Here, we solved a 1.94 Å X-ray crystal structure of *Bacteroides fragilis* CASDH by molecular replacement. BfCASDH is composed of three domains with a fold similar to saccharopine dehydrogenase but with a distinct active site arrangement. Using steady-state methods, we determined *k*_*cat*_ and *k*_*cat*_*/K*_*m*_ for BfCASDH and *Clostridium leptum* CASDH using putrescine, diaminopropane, aspartate semi-aldehyde, NADH, and NADPH as substrates. These data revealed evidence of cooperativity in BfCASDH. Putrescine is the likely polyamine substrate and NADPH is the coenzyme used to complete the reaction, forming carboxyspermidine as a product. These data provide the first kinetic characterization of CASDH—a key enzyme in the production of microbial polyamines.

Polyamines have been shown to modulate an array of biological processes including cell proliferation, the regulation of gene expression, and bacterial biofilm formation such that polyamines are considered an essential component of living organisms (1, 2). Humans and most other eukaryotes biosynthesize the polyamine spermidine from the precursors ornithine and S-adenosyl-L-methionine (SAM) in a three-enzyme pathway. Ornithine decarboxylase converts ornithine to putrescine (a 1,4-diaminobutane polyamine), SAM decarboxylase removes the methionine carboxylate of SAM, and spermidine synthase catalyzes the transfer of the aminopropyl moiety from decarboxy-SAM to putrescine forming spermidine (3). In addition to endogenous biosynthesis, epithelial cells of the human alimentary tract can uptake exogenous polyamines found in digested food or produced by gut microbes (4, 5). The dysregulation of spermidine production in the gut has been associated with tumor progression in pancreatic ductal carcinoma (6), colorectal carcinoma (7, 8, 9), and, conversely, spermidine supplementation has been shown to protect mice from colorectal carcinogenesis (10). The link between exogenous spermidine production by gut microbes and the modulation of human health justifies an investigation of polyamine metabolic pathways found in human gut microbes. In this study, we investigated the largely uncharacterized carboxyspermidine dehydrogenase that produces carboxyspermidine as a precursor to the polyamine spermidine in an alternative polyamine biosynthetic pathway first identified in *Vibrio alginolyticus* (11, 12). This alternate pathway is known to be present in many bacterial species including a majority of the most prevalent human gut microbes (13, 14, 15).

Carboxyspermidine dehydrogenase (CASDH – E.C. 1.5.1.43) was first shown to produce carboxynorspermidine through the reductive condensation of 1,3-diaminopropane and aspartate semi-aldehyde using NAD(P)H as a coenzyme (Fig. 1*A*). The carboxynorspermidine product was subsequently decarboxylated by carboxyspermidine decarboxylase to form norspermidine. This activity was demonstrated in *V. aglinolyticus*, a marine bacterium, in the presence of NADH or NADPH using gas chromatography to identify the derivatized carboxynorspermidine product (12). Accordingly, CASDH is also referred to as carboxynorspermidine dehydrogenase (CANSDH) in the literature. Two additional studies used HPLC separation to identify CANSDH activity in *Vibrio cholera* (13) and *Campylobacter jejuni* (14) and to establish the widespread presence of this route to spermidine in many bacterial phyla. A bioinformatic survey revealed the prominence of this pathway in human gut microbes; indeed 75% of 32 of the most prevalent species encode genes predicted to have CASDH and CASDC (carboxyspermidine decarboxylase) activity (15).

**Figure 1:**
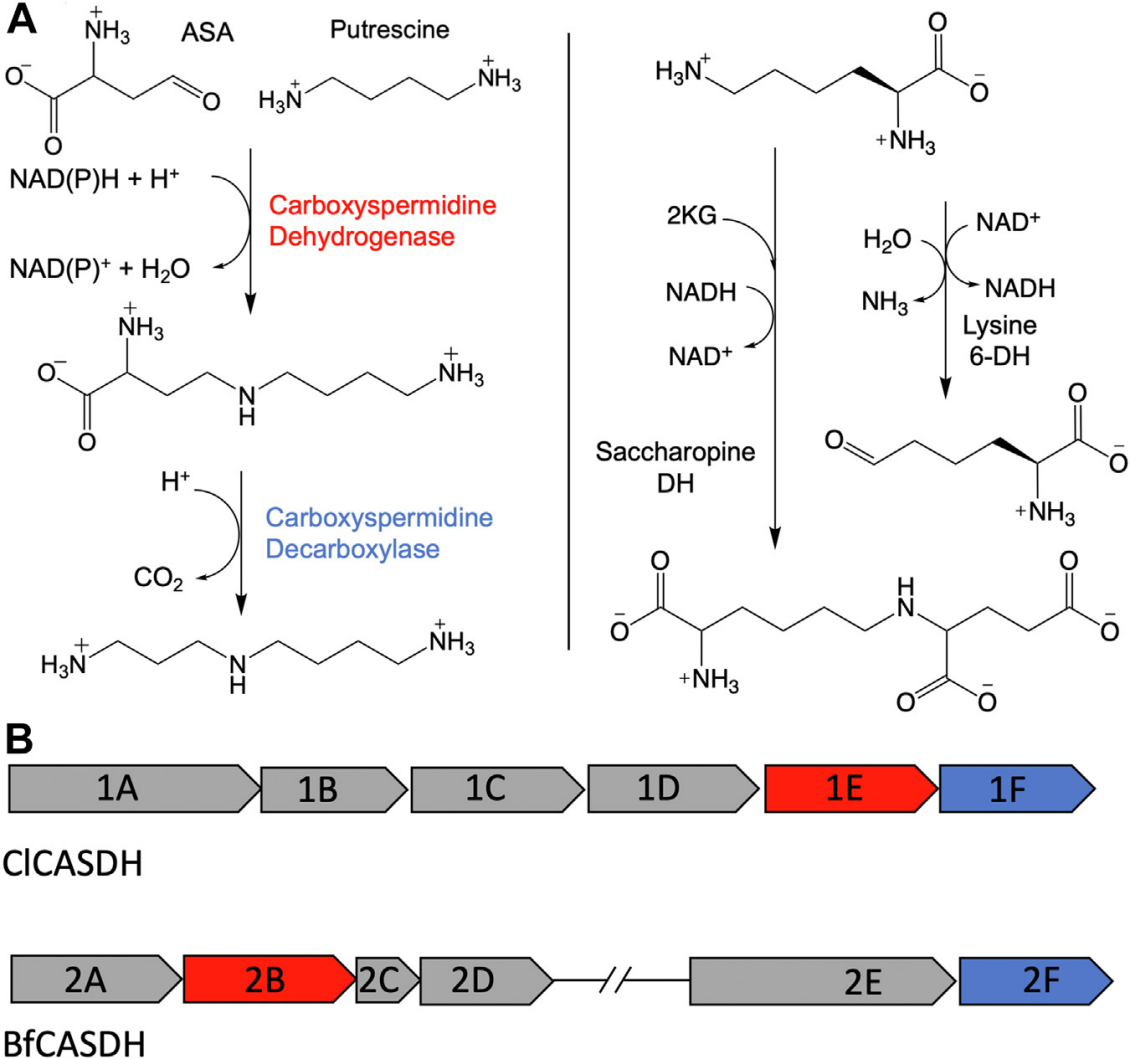
**Spermidine biosynthetic schemes and operons.***A*, Schemes. (*Left*) spermidine biosynthesis; (*right*) saccharopine and lysine 6-dehydrogenase biosynthesis (2 KG – α-ketoglutarate; DH – dehydrogenase). *B*, ClCASDH and BfCASDH operons. CASDH (*red*). CASDC (*blue*). SAM decarboxylase (1A), arginine decarboxylase (1B), spermidine synthase (1C), agmatine uerohydrolase (1D). Uncharacterized protein believed to be involved in DNA repair and recombination (2A), Bacterioferritin co-migratory protein (2C), RecA DNA repair protein involved in homologous recombination (2D). UvrD helicase is involved in excision and mismatch repair (2E).

CASDH is a homolog of enzymes that perform similar chemistry including saccharopine dehydrogenase, L-lysine 6-dehydrogenase, and homospermidine synthase. (Fig. 1*A*). The relationship between these homologs has been discussed in previous literature, but the sequence determinates that distinguish CASDH chemistry remained experimentally undefined (16, 17). We hypothesized that principal constituents of the gut flora would use CASDH and CASDC to produce spermidine, using putrescine as a substrate, as norspermidine is found in low concentrations in the gut lumen and many species cannot biosynthesize 1,3-diaminopropane (Fig. 1*A*). We chose candidates from microbial species representing the two most prominent gut phyla: *Bacteroides fragilis* and *Clostridium leptum.* Both species encode predicted CASDH and CASDC genes. *C. leptum* encodes CASDH and CASDC alongside a predicted SAM decarboxylase and spermidine synthase suggesting the presence of two paths for spermidine production. Interestingly, in *B. fragilis*, CASDH and CASDC are found on separate operons that each contain additional genes associated with homology-directed DNA repair (Fig. 1*B*). The polyamines putrescine, spermidine, and spermine have recently been demonstrated to stimulate RAD51-mediated DNA strand exchange in a mouse hair follicle model (18). The BfCASDH operon constituents may have a similar function.

We heterologously expressed and purified CASDH from both species. Steady-state kinetic analysis was performed to investigate substrate specificity but also revealed cooperativity between the two active sites of dimeric BfCASDH. The solution of a 1.94 Å BfCASDH X-ray crystal structure, with NADP^+^ bound, allowed a characterization of CASDH domain structure and active site arrangement, as well as a comparison with homolog enzymes from the saccharopine dehydrogenase, L-lysine 6-dehydrogenase, and homospermidine synthase families.

## Results and discussion

### CASDH substrate specificity

To investigate substrate specificity for BfCASDH and ClCASDH, both enzymes were heterologously expressed and purified from *E. coli* BL21 (DE3) cells using nickel-chelating sepharose affinity chromatography (Fig. S1). Aggregation was visible for both enzymes when concentrating above 2 mg/ml. The addition of 1 mM dithiothreitol to the storage buffer prevented aggregation and allowed concentration to 10 mg/ml.

Steady-state kinetic parameters were determined in the presence of NADPH, NADH, DAP, putrescine, and ASA to define the substrate specificity for both BfCASDH and ClCASDH (Table 1). Decreasing absorbance at 340 nm was measured as the oxidation of NAD(P)H proceeded. Parameters were determined by fitting secondary plots of initial rates using the Michaelis-Menten equation (Equation 1 – BfCASDH with NADPH; ClCASDH with all substrates) or the Hill equation (Equation 2 – BfCASDH with ASA, DAP, and putrescine).

**Table 1:** Kinetic parameters

Enzyme	Initiating substrate	*k*_cat_ (s^−1^)^a^	*K*_m_ (mM)^a^	*k*_cat_/*K*_m_ (M^−1^s^−1^)	Hill Coefficient	*K*_d_ (mM)
BfCASDH	Putrescine	0.41 ± 0.02	4.2 ± 0.3	96 ± 7	2.1 ± 0.2	11 ± 1
BfCASDH	DAP	0.34 ± 0.03	19.7 ± 3.6	17 ± 3	1.8 ± 0.1	15 ± 2
BfCASDH	ASA	0.64 ± 0.02	2.7 ± 0.1	230 ± 15	1.5 ± 0.1	-
BfCASDH	NADPH	0.39 ± 0.05	0.057 ± 0.003	6700 ± 200	-	-
ClCASDH	Putrescine	0.37 ± 0.01	5.0 ± 0.6	74 ± 10	-	-
ClCASDH	DAP	0.49 ± 0.03	6.9 ± 1.1	71 ± 12	-	-
ClCASDH	ASA	-	-	260 ± 20	-	-
ClCASDH	NADPH	0.22 ± 0.02	0.016 ± 0.002	13,800 ± 900	-	-

a *k*_cat_ and *K*_m_ values should be considered apparent as ASA concentrations were subsaturating.

**Figure 2:**
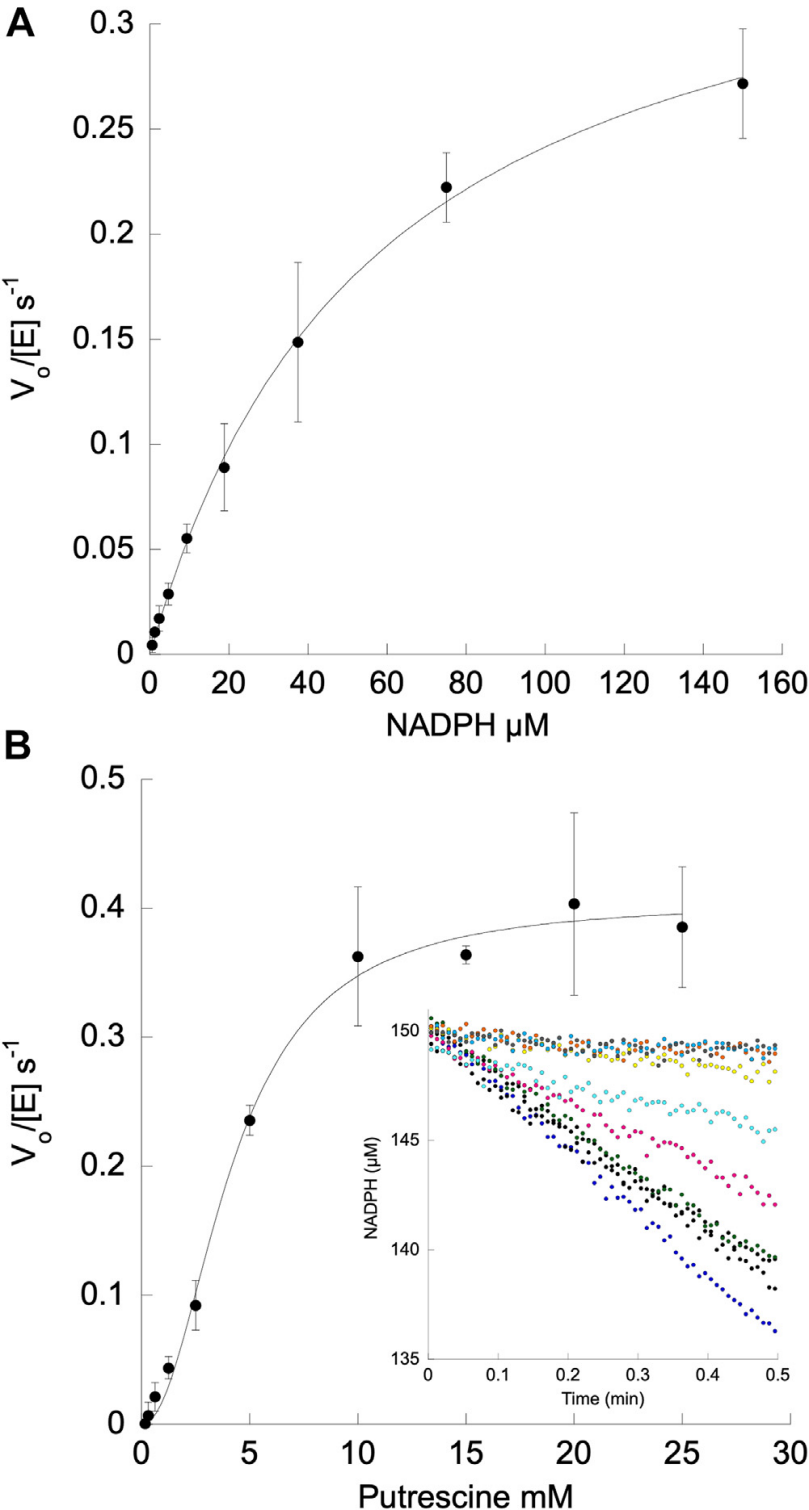
**BfCASDH steady-state kinetic plots.** Initial rates were determined by applying linear fits to primary plots of NAD(P)H oxidation (ε = 6220 M^−1^ cm^−1^). Secondary plots were fit with the Michaelis-Menten (*A*) or Hill equation (*B*). Error bars represent the standard deviation of at least three trials. *A*, one micrometre BfCASDH, 2.5 mM ASA (aspartate semi-aldehyde), 20 mM putrescine, and varied NADPH. *B*, one micrometre BfCASDH, 2.5 mM ASA, 150 μM NADPH and varied putrescine. The buffer was 25 mM potassium phosphate, pH 7.5. Inset – example raw kinetic data for varied putrescine.

Both BfCASDH and ClCASDH selected NADPH over NADH. NADH initial rates were within the limit of detection for both enzymes (<0.008 *V*_O_/[E] s^−1^) (Figs. 2*A*, S3*D*, and S5, *D* and *E*). BfCASDH and ClCASDH have similar *k*_*cat*_ values for putrescine and DAP (PUT *k*_*cat*_ = 0.41 s^−1^ ± 0.02 and 0.37 ± 0.01 respectively; DAP *k*_*cat*_ = 0.34 s^−1^ ± 0.03 and 0.49 ± 0.03 respectively) (Table 1; Figs. 2*B*, S3C, and S5, *B* and *C*). At ASA concentrations above 2.5 mM, significant inhibition was observed for both Bf- and ClCASDH (Fig. S3*B*). We attempted to fit these data to account for this apparent substrate inhibition, but the rapid decline in the initial rate precluded a satisfactory fit. The inhibition for ClCASDH was particularly sharp above 2.5 mM preventing estimation of *k*_*cat*_ for ASA. The significance of inhibition *in vivo* at a 2.5 mM concentration is unclear.

A modified form of the Michaelis-Menton equation solved in terms of *k*_*cat*_*/K*_*m*_ (rather than *K*_*m*_) was used to derive each *k*_*cat*_*/K*_*m*_ value (Equation 3) (19). BfCASDH had a *k*_*cat*_*/K*_*m*_ 21-fold higher for NADPH than for ASA (6700 M^−1^s^−1^ ± 200 *versus* 230 M^−1^s^−1^ ± 15). For ClCASDH this difference was 53-fold (13,800 M^−1^s^−1^ ± 900 *versus* 260 M^−1^s^−1^ ± 20). The *k*_*cat*_*/K*_*m*_ difference was a further 2.5-fold and 3.4-fold greater when comparing NADPH and putrescine. BfCASDH has a PUT *k*_*cat*_*/K*_*m*_ 5.6 times higher than for DAP (96 ± 7 M^−1^s^−1^ *versus* 17 ± 3 M^−1^s^−1^). ClCASDH *k*_*cat*_*/K*_*m*_ values for PUT and DAP were 74 ± 10 M ^−1^s^−1^ and 71 ± 12 M^−1^s^−1^. These data suggest that both enzymes can use putrescine or DAP and thus produce carboxyspermidine or carboxynorspermidine.

To consider the potential availability of putrescine or DAP within *B. fragilis* or *C. leptum* cells, a bioinformatic analysis was used to identify the presence of the enzymes necessary to biosynthesize putrescine and DAP. In bacteria, aspartate is converted to L-4-aspartyl phosphate by aspartate kinase. L-4-aspartyl phosphate is converted to ASA by L-aspartate semialdehyde dehydrogenase, an enzyme encoded by both *B. fragilis* and *C. leptum*, indicating the availability of ASA as an aminopropyl donor. We used the UniProt database to find homolog query sequences for the enzymes necessary to make putrescine and DAP. Putrescine is produced by arginine decarboxylase and agmatine ureohydrolase, which are present in both species. DAP is produced from ASA in a 2-step process using diamino butyrate aminotransferase (DABA AT) and diamino butyrate decarboxylase (DABA DC) (14). Query sequences were selected among homologs based on the level of characterization and species similarity with the organisms in this study. *Rhizobium meliloti* and *Clostridium putrefaciens* sequences were used for DABA AT and *Bacteroides coprosuis* and *Clostridium cochlearium* sequences for DABA DC. *B. fragilis* has a DABA AT but not a DABA DC homolog. *C. leptum* has neither DABA AT or DC. This suggests that putrescine is the biologically available substrate and that carboxyspermidine is the CASDH product for both species, but the potential for uptake and incorporation of exogenous DAP is also possible.

### BfCASDH cooperativity

Secondary plots from reactions with varied NADPH were fit using the Michaelis-Menten equation (Fig. 2*A* and Equation 1). When ASA, putrescine, and DAP were varied, a sigmoidal approach to *V*_*max*_ was observed. These data were fit using the Hill equation (Fig. 2*B* and Equation 2; Fig. S3, *A* and *C*). Thus, cooperativity is evident for ASA, putrescine, and DAP but not NADPH. To investigate whether binding alone was cooperative, we measured the binding of putrescine and DAP by BfCASDH by observing a decrease in intrinsic tryptophan fluorescence emission at 340 nm. Both putrescine and DAP bind weakly with *K*_*d*_ values of 11 mM ± 1 and 15 mM ± 2, respectively, which are comparable to the measured *K*_*m*_ values (Fig. S4, *A* and *B*). In each case, the data were best fit using the sigmoidal rather than the hyperbolic form of the binding isotherm equation (Equation 4) suggesting that binding alone is cooperative.

### BfCASDH structure determination

We determined a crystal structure of BfCASDH by X-ray crystallography at a resolution of 1.94 Å. A PDB sequence search was used to identify candidate molecular replacement models for structure solution. PDB 4RL6, a *Streptococcus pneumoniae* protein annotated as a saccharopine dehydrogenase, without an associated literature publication, and deposited by the Northeast Structural Genomics Consortium, had 57.7% identity (Clustal Omega) with BfCASDH. Phenix Phaser (20, 21) completed molecular replacement using the 4RL6 model with an LLG of 1895.88 and a TFZ score of 46.0. Phenix Autobuild placed 87% of BfCASDH residues with a final R_free_ value of 25.2. Electron density corresponding to NADP^+^ was clearly visible upon initial inspection of the 2m*F*_*o*_–D*F*_*c*_ maps following molecular replacement. Data collection and refinement statistics are found in Table 2. These data have been deposited with the PDB with accession number 8DEB.

**Table 2:** Data collection and refinement statistics

Parameters	BfCASDH-NADP^+^
Data collection	
Spacegroup	P2_1_2_1_2_1_
Unit cell (Å)	a = 108.23
	b = 85.81
	c = 98.21
Resolution range (Å)	39.88–1.94
Completeness (%)	99.4 (92.4)
Total reflectons	894,708
Unique reflections	67,435
*I*/σ	10.4 (2.1)
*R*_pim_^a^	5.4 (42.1)
CC ½^c^	99.8 (70.4)
CC∗^c^	99.9 (90.9)
Multiplicity	13.3 (12.4)
Refinement	
Resolution range (Å)	39.88–1.94
No. of reflections	67,304
*R*_work_/*R*_free_^b^	17.86/20.87
No. non-hydrogen atoms	6718
Protein atoms	6188
Ligand atoms	152
Waters	434
Ramachandran favored (%)	97.91
Ramachandran outliers (%)	0.0
Wilson *B*	23.05
Average *B* (Å^2^)	27.76
Protein	27.56
Ligand/ion	26.15
R.m.s. deviations	
Bond lengths (Å)	0.003
Bond angles (°)	0.62

Data were collected on beamline 9-2 at the Stanford Synchrotron Radiation Lightsource. Values in parentheses are for the highest resolution shells.

a *R*_pim_ = $\sum_{hkl}\sqrt{1/n-1}\left|I_{hkl}-{\left\langle I\right\rangle }_{hkl}\right|/\sum_{hkl}I_{hkl}$ where n is the multiplicity of related reflections.

b CC∗ = $\sqrt{\frac{2{CC}_{1/2}}{1+{CC}_{1/2}}}$ where ${CC}_{1/2}$ = CC for random half sets (40).

c *R* = $\sum \left|F_{o}-\left|F_{c}\right|\right|/\sum \left|F_{o}\right|$ where $F_{o}$ = to the observed structure factors and $F_{c}$ = structure factors calculated from the model. 2.97% of the reflections were initially reserved to create a *R*_free_ test set used during each subsequent round of refinement.

### BfCASDH structure and assembly

**Figure 3:**
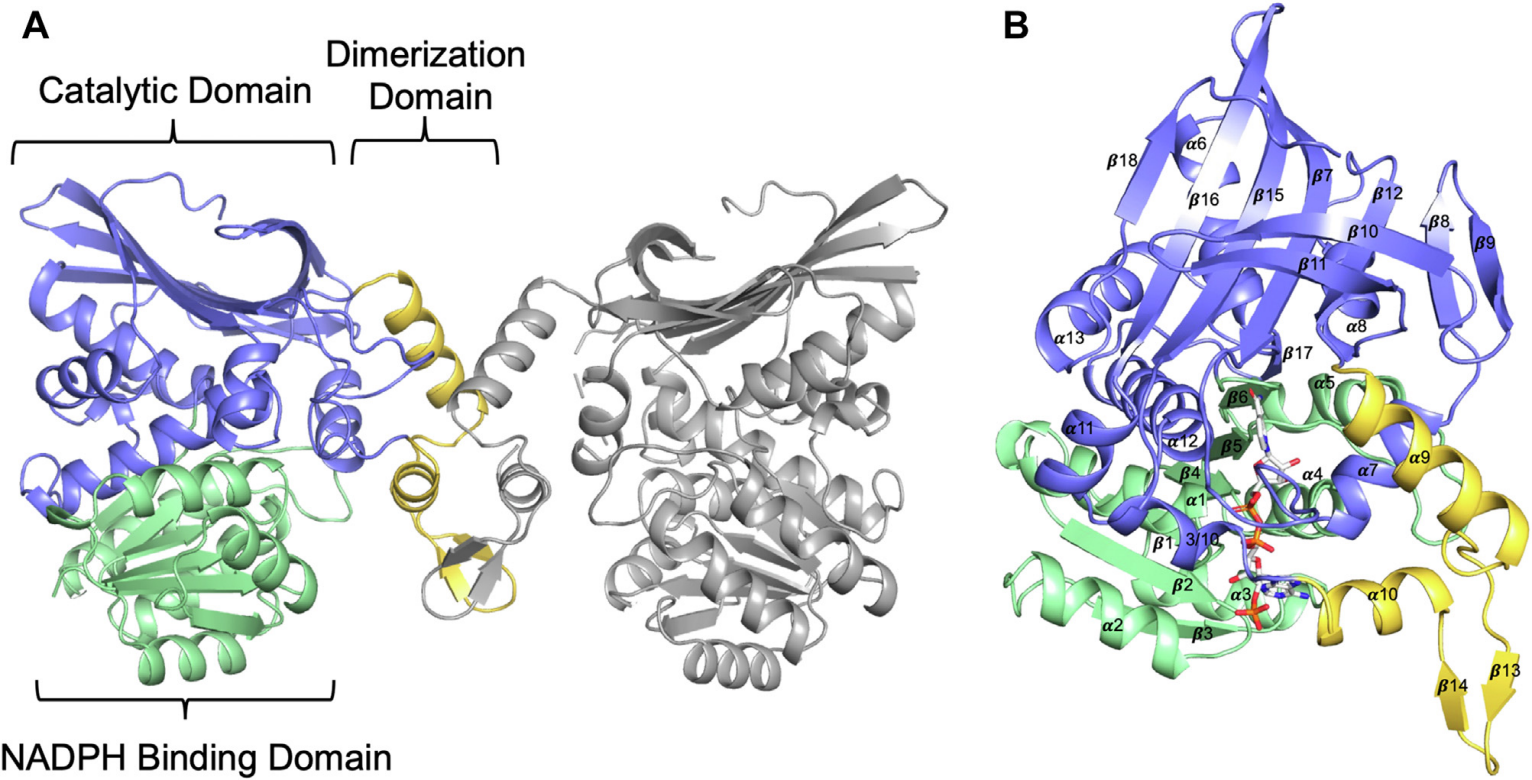
**BfCASDH structural overview.***A*, BfCASDH dimer. *B*, Secondary structure labeling. Chain A monomer is shown. NADPH - *sticks* with *gray* carbons. NAD(P) binding domain – *green*. Catalytic domain – *blue*. Dimerization domain – *yellow*.

The BfCASDH asymmetric unit contained two monomers stabilized by an extensive network of hydrogen bonds bridged by water molecules, representing a crystallographic interface. Inspection of a two-fold rotational symmetry mate by PDBePISA (22) revealed a 1010 Å^2^ surface with a single salt bridge between Arg259 and Glu253 and extensive hydrophobic packing between the monomers involving a total of 27 amino acids. This interface forms a probable biologic dimer that is structurally similar to the PDBePISA-predicted dimer for 4RL6 and to proteins from the saccharopine dehydrogenase and lysine-6-dehydrogenase families as described below (Fig. 3*A*). Size exclusion chromatography was performed using a HiLoad 16/600 Superdex 200 column. BfCASDH retention time corresponded to a molecular weight of 88.4 kDa, equivalent to a dimer of the 45.1 kDa BfCASDH monomers. ClCASDH eluted with a retention time corresponding to a molecular weight of 107.1 kDa which is 2.4X the monomeric weight of 44.6 kDa. These data suggest that both enzymes are biological dimers (Fig. S2).

**Figure 4:**
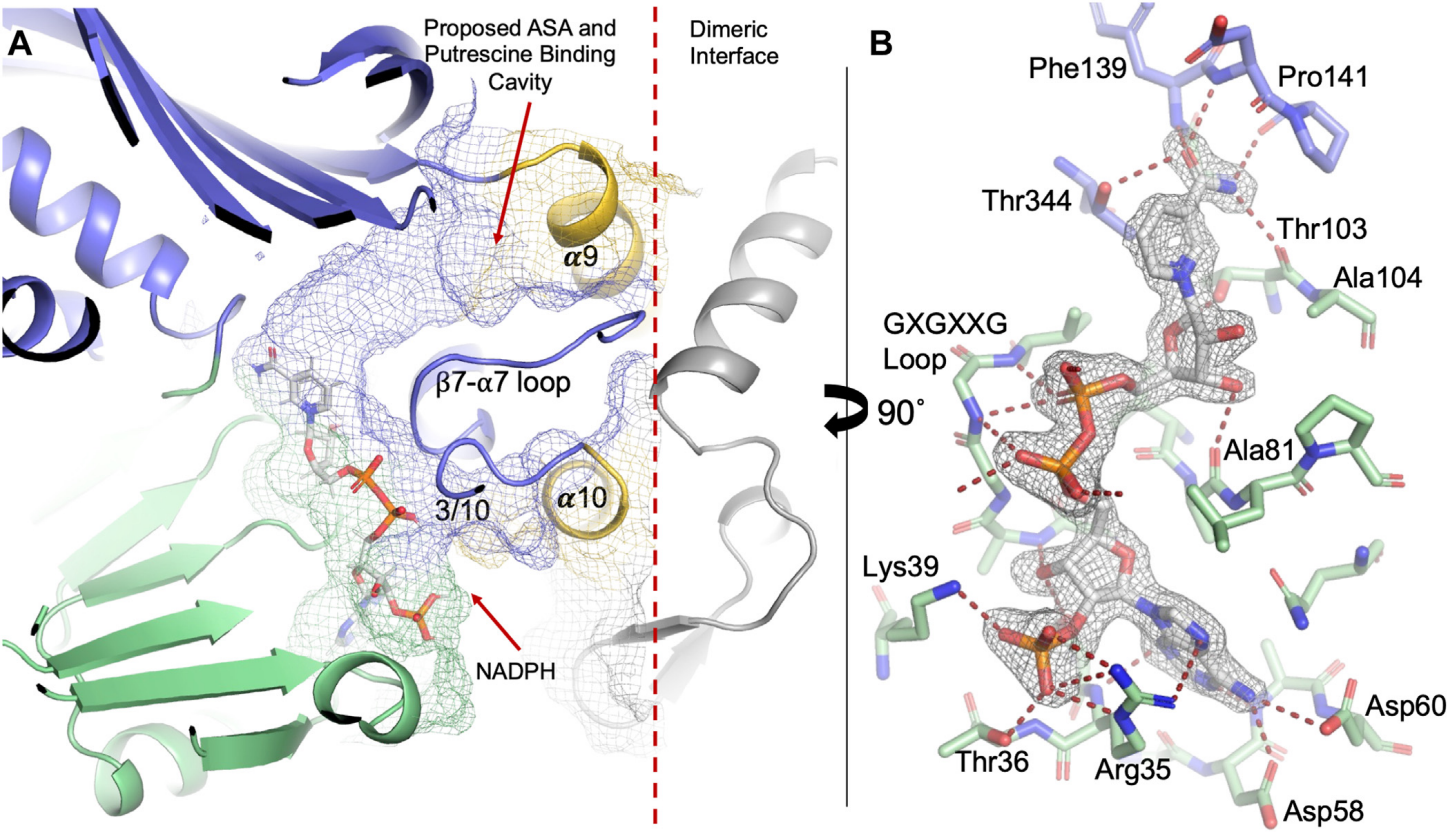
**BfCASDH NADPH binding site.***A*, BfCASDH has an active site tunnel (shown as mesh generated by the Computer Atlas of Surface Topography – CASTp) that extends across all three domains and contains a bound NADP^+^ molecule in each chain. *B*, NADP^+^ is bound to a GXGXXG loop in a Rossmann fold NAD-binding domain with the C2′ phosphate coordinated by multiple hydrogen bonds (NADP^+^ from chain B is shown. Chain A has a slightly weaker density around C4 of the nicotinamide ring). Gray mesh is a 2m*F*_*o*_–*DF*_*c*_ electron density map contoured at 1.5 σ.

BfCASDH is composed of three domains (Fig. 3, *A* and *B*). An NAD(P)-binding domain spans residues 1 to 136 and is a canonical Rossmann-fold domain with 321456 β-strand topology encoding a GXGXXG loop, from residues 8 to 13, that stabilizes the diphosphate backbone of NADP^+^. The catalytic domain, from residue 137 to 250 and 290 to 397 is an α/ β domain composed of ten β-strands, six α-helices, and one 3/10 helix. The catalytic domain and NAD(P)-binding domain associate *via* an extended interface formed by α 12 and β 17, with β 17 contributing one additional parallel strand to the NAD(P)-binding domain’s β sheet. A dimerization domain interrupts the catalytic domain from residue 251 to 289 and is composed of two α helices and two β strands that form a small antiparallel sheet. The dimerization domain is fully resolved in both chains but is adjacent to the two sequences from the chain B catalytic domain that were disordered, and left unmodeled, in our structure. The 3/10 helix of the catalytic domain is disordered from residue 290 to 299 and connects to α 10 of the dimerization domain. Additionally, α 9 of the dimerization domain overlays a long loop between β 7 and α 7 of the catalytic domain. The β 7-α 7 loop is disordered from 172 to 180. The disordered 3/10 helix and β 7-α 7 loop form a cap over the NADP^+^ binding channel, as visible in chain A (Fig. 4*A*). The flexibility of the 3/10 helix and β 7-α 7 loops are likely to play a role in conformation changes during the catalytic cycle. The proximity of the dimerization domain, and our observation of cooperative enzyme kinetics, suggests communication with structural elements of the catalytic domain that overlay the active site and across the dimeric interface. A DynDom (23) analysis of domain motion between chains A and B reveals a small, 3.2° rotation of the dimerization domain in chain B over the active site formed by the catalytic and NADP-binding domains. Interestingly, this 3.2° rotation and “closure” in chain B co-occurs with the disorder of the segments from the catalytic domain described above.

### NADP binding

Kinetic analysis revealed no evidence of BfCASDH turnover with NADH as a co-substrate. We were unable to co-crystallize BfCASDH with NAD^+^. BfCASDH was co-crystallized with NADP^+^ and complete density for NADP^+^ was evident following molecular replacement in both chains A and B (Fig. 4*A*). The descriptions below refer to chain B but include the slight differences observed in chain A. The hydrogen bonding network surrounding the C2′-phosphate of the 5′-phosphate adenosine-2′,5′-bisphosphate moiety suggests a basis for NADP selectivity for BfCASDH (Fig. 4*B*). The guanidino group of Arg45 hydrogen bonds with two of the phosphoryl oxygens. The hydroxyl from the Thr36 forms a third hydrogen bond with the phosphoryl group. Water 339 forms a fourth hydrogen bond and is stabilized within a pocket by hydrogen bonds formed with Thr36 and Ser34. A fifth hydrogen bond was modeled to the omega nitrogen of Lys39. Chain A has incomplete density for the omega nitrogen of Lys39, though we modeled it using the same rotamer. The adenosine is in the *anti*-conformation and is stabilized by six hydrogen bonds. N6, N3 and N1 form hydrogen bonds with Asp60, Ala59, Asp58 and Arg35. N6 and N7 are also stabilized by hydrogen bonds to waters 205 and 164. Both ribose sugars are in a C2′-*endo* conformation. The adenosine ribose C3′ hydroxyl hydrogen bonds with the Ser34 hydroxyl and the Ala9 amine. The C3′ hydroxyl of the ribose sugar from the ribosylnicotinamide hydrogen bonds with the carbonyl oxygen of Ala81 and the C2′ hydroxyl is not hydrogen bonded. The 5′-phosphates of the pyrophosphate linkage hydrogen bond along the GXGXXG loop as is typical for Rossmann-fold domains. The nicotinamide ring is in the *anti*-conformation with complete density when viewed using a *2F*_*o*_*-F*_*c*_ map contoured at 1.5 σ in chain B but with a slight break in density between C3 and C7 of the amide group in chain A. The amide N is stabilized through hydrogen bonds with the backbone carbonyl oxygens of Thr103 and Pro141. The amide oxygen is stabilized through hydrogen bonds with the amines of Phe139 and Asp140. The nicotinamide ring is stabilized by van der Waals packing with Val12 and Thr344. In this conformation, the pro-*R* hydride would be directed toward the open active site suggesting that BfCASDH performs a pro-*R* specific hydride transfer which is consistent with NAD(P)-dependent enzymes that bind NAD(P) with the nicotinamide ring in the *anti-*conformation (24).

### BfCASDH active site

**Figure 5:**
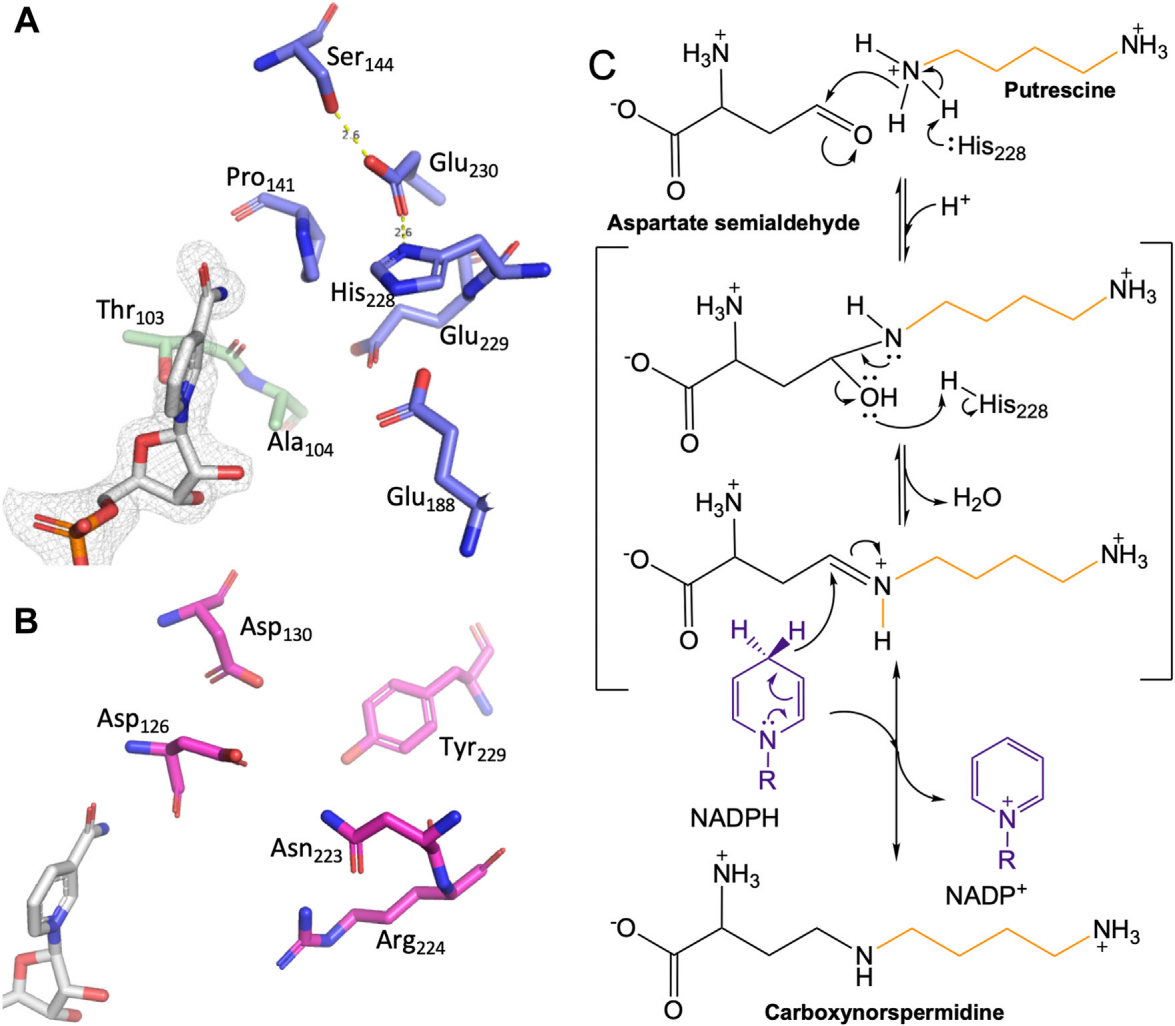
**BfCASDH active site analysis.***A*, BfCASDH active site. NADPH – gray sticks (mesh is a 2m*F*_*o*_–*DF*_*c*_ electron density map contoured at 1.5 σ). Ser144, Glu230 and His228 are within hydrogen bonding distance and His228 is positioned to act as an active site base. Glu229 and Glu188 may act to coordinate and position substrate functional groups. *B*, saccharopine dehydrogenase (1E5Q) from the pathogenic fungus *Magnaporthe grisea* is a close structural relative of BfCASDH with a DALI rmsd of 2.8 Å (370 of 449 residues aligned), yet the active site arrangement differs significantly. *C*, proposed catalytic mechanism for carboxyspermidine synthesis.

NADP^+^ is positioned in a V-shaped active site with two entrance channels, one through the NAD(P)-binding domain and the other through the catalytic domain and flanked by α 9 of the dimerization domain (Fig. 4*A*). NADPH appears to enter through the NAD(P)-binding channel which positions the nicotinamide ring at the apex of the V, suggesting that ASA and putrescine bind by entering the channel within the catalytic domain. Across from the nicotinamide ring, the active site is flanked by Glu188, Glu229, and His228 (Fig. 5*A*). Glu188 and Glu229 sit deep within the active site and are positioned to coordinate ASA as it binds adjacent to the nicotinamide ring. His228 is positioned to serve as a general base in the catalytic cycle. It is within hydrogen bonding distance of Glu230 which is within hydrogen bonding distance of Ser144. A series of well-resolved water molecules form a chain from Ser144 to the surface of BfCASDH. These observations suggest a path for proton exchange during catalysis.

The arrangement of the active site residues leads us to propose a catalytic mechanism for CASDH (Fig. 5*C*). The *k*_*cat*_*/K*_*m*_ values for ASA and putrescine are much lower than for NADPH and cooperativity was only observed for ASA and putrescine. This suggests independence between NADPH and ASA and putrescine binding, although the possibility that NADPH must bind first cannot be ruled out. ASA likely binds before putrescine as suggested by the active site arrangement. His228 is positioned to act as a general base, activating a primary amine of putrescine for nucleophilic attack. Two protons are necessary for the hydration of the carbonyl oxygen of ASA prior to water release. His228 could donate both protons, transferring the first from putrescine and contributing a second through the His-Glu-Ser relay. Following water release, the pro-*R* hydride of the dihydronictoinamide ring reduces the Schiff base to form NADP^+^ and carboxyspermidine.

### Structural homologs of BfCASDH

A DALI search using BfCASDH as the query structure reveals PDB 4RL6 as the nearest homolog with an RMSD of 1.8 Å over 389 of 414 residues. PDB 4INA is similar with an RMSD of 2.2 Å over 387 of 397 residues. Both 4RL6 and 4INA are annotated as saccharopine dehydrogenases but were solved by the Northeast Structural Genomics Consortium and have no associated publication or functional characterization. The String database (25) was used to examine the genome neighborhoods for the 4RL6 and 4INA genes and provides evidence that both are CASDH enzymes, not saccharopine dehydrogenases. 4RL6 is in an operon with genes predicted to encode CASDC, spermidine synthase, agmatine deiminase, and ureohydrolase activities that are associated with polyamine biosynthesis. 4INA is in an operon containing a predicted CASDC (suggesting a spermidine or norspermidine product) along with a malic enzyme, a 16s rRNA methyltransferase, and several uncharacterized membrane-associated proteins. Polyamines have known roles in the regulation of translation, although the exact role of this operon is unknown (26). This bioinformatic analysis suggests that both 4RL6 (UniProt: A0A0H2ZP94) and 4INA (UniProt: Q7MSS8) are misannotated and are CASDH enzymes involved in polyamine biosynthesis.

**Figure 6:**
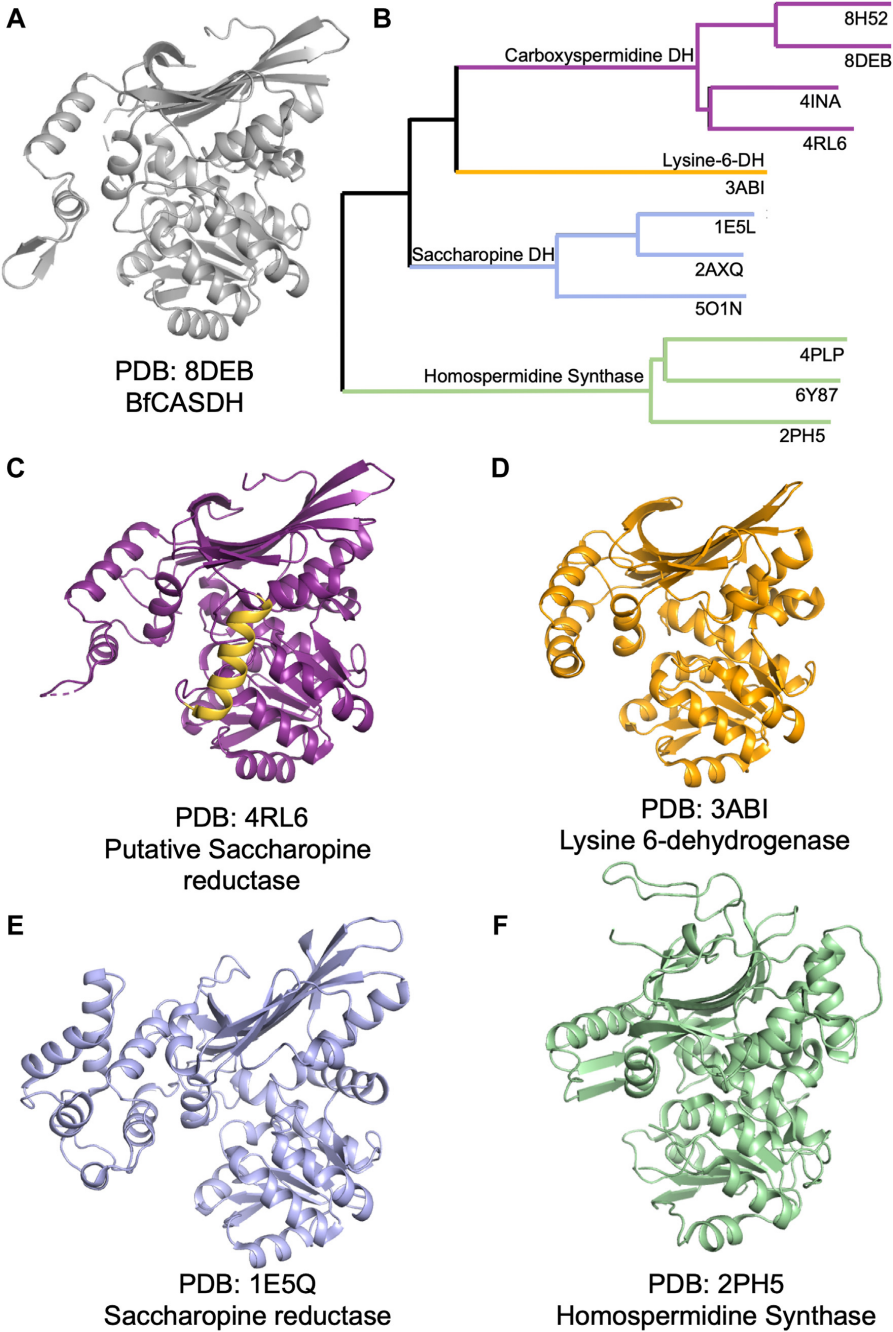
**BfCASDH structural homologs.***A*, BfCASDH monomer. *B*, dendrogram generated by DALI using the top ten structural homologs of BfCASDH. *C*–*F*, four example structural homologs predicted by DALI and aligned with BfCASDH chain B (*gray*). The *yellow* helix in 4rl6 is not present in BfCASDH.

The next three nearest DALI matches were 3ABI (2.3 Å r.m.sd; 337 of 349 amino acids aligned) which is a L-lysine 6-dehydrogenase (27), 1E5Q (2.8 Å r.m.sd; 370 of 449 amino acids aligned) which is a saccharopine dehydrogenase (reductase) (28), and 4XR4 (3.1 Å r.m.sd; 358 of 474 amino acids aligned) which is a homospermidine synthase (29). An overlay of these three structures with BfCASDH using the cealign algorithm in PyMol demonstrates a similar fold for the NAD(P)-binding and catalytic domains, but differences in the dimerization domain and differences in active site residues (Figs. 5*B* and 6, *A*–*F* and S6, *A*–*E*). We performed kinetic assays to investigate the possibility that our CASDH enzymes could catalyze saccharopine or L-lysine-6 DH activity as these are the two most closely related homologs. We assayed 1 μM Bf- and ClCASDH with L-lysine in the presence of NAD^+^ or NADP^+^ (L-lysine-6 DH activity) and L-lysine and α-ketoglutarate in the presence of NADH or NADPH (saccharopine DH activity) (Scheme S1). In all experiments except one, turnover was within error of the limit of detection of our assay (<0.008 *V*_O_/[E] s^−1^) (Table S1). BfCASDH showed slight activity toward the production of saccharopine in the presence of NADPH (0.039 s^−1^ ± 0.006). Although these measurements were above the detection limit, the rate was 16-fold lower than the *k*_cat_ measured for carboxyspermidine production. These data provide additional evidence that Bf- and ClCASDH specifically produce carboxyspermidine despite their structural similarity to enzymes performing similar chemistries.

After we had drafted this article, Kyung Yeol Ko *et al.* (30) published structures of CASDH from *Helicobacter pylori* (HpCASDH) at 2.2 Å (apo), 2.9 Å (apo) and 3.1 Å (NADP bound). A DALI comparison with BfCASDH calculates a 2.0 Å RMSD (376 of 397 residues aligned) for HpCASDH – apo and a 0.90 Å RMSD (384 of 397 residues aligned) for HpCASDH – NADP^+^. These structures show a significant conformational change upon NADP^+^ binding as discussed by the authors. A DynDom analysis calculates a 10.2° closure when comparing HpCASDH – apo and HpCASDH – NADP^+^. This closure primarily involves movement of the α 10 and β 13 to 14 loop of the dimerization domain. This closure may also occur in BfCASDH. If so, it would be expected to precede ASA and putrescine binding.

A comparison the BfCASDH active site with structural homologs reveals significant differences in active site arrangement. The saccharopine dehydrogenase from *Magnaporthe grisea* (1E5Q; Fig. 5*C*) and the L-lysine-6-dehydrogenase from *Pyrococcus horikoshii* (3ABI, Fig. S6) do not have the His228, Glu229, Glu230 sequence that appears central to the CASDH catalytic cycle (Fig. S7), but both 4RL6 and 4INA conserve this triad and other active site features lending support to our hypothesis that they are misannotated CASDH enzymes and that the CASDH family forms a distinct group sharing a common ancestor with enzymes of the saccharopine dehydrogenase and homospermidine synthase families.

## Conclusion

Carboxyspermidine dehydrogenase, from two major gut microbes, performs the reductive condensation of aspartate semi-aldehyde (ASA) and putrescine using NADPH as a coenzyme, in the formation of carboxyspermidine. CASDH from *C. leptum* and *B. fragilis* both have significantly slower *k*_*cat*_*/K*_*m*_ values for ASA and putrescine than for NADPH and the active site structure suggests that NADPH binds rapidly but independently of ASA and putrescine. Kinetic and binding cooperativity observed for ASA and putrescine in BfCASDH indicates the presence of structural communication across the dimeric interface. Our structural analysis of BfCASDH suggests a misannotation of two putative saccharopine dehydrogenases (PDB: 4RL6 and 4INA). Both appear to be CASDH enzymes.

## Experimental procedures

### Preparation of overexpression plasmids

Gene sequences for the *B. fragilis* and *C. leptum* strains of carboxyspermidine dehydrogenase were obtained from the NCBI database with accession numbers WP_005801052.1 and EDO61991.1. The sequences were codon optimized for expression in *E. coli* by GenScript and ligated into pET-28b vectors containing an N-terminal hexahistidine affinity tag. The resulting plasmids were transformed into New England Biolabs BL21 (DE3) cells for expression.

### Aspartate semialdehyde synthesis

Aspartate semialdehyde was synthesized following previously published methods (31, 32, 33). In short, the reduction of the Weireb amide derived from commercially available a-tert-butyl-(S)-N-tert-butoxycarbonylaspartate with DIBAL followed by global deprotection with trifluoroacetic acid (TFA) led to a good yield of aspartate semialdehyde as its TFA salt.

### Protein expression and purification—*B. fragilis*

CASDH was expressed in a baffled flask with 1 L of LB Miller broth containing 50 μg/ml kanamycin inoculated with 10 ml of overnight culture and grown to A_600_ of 0.6 to 0.8 at 37 °C (∼3 h). The cultures were induced with 200 μl 1 M IPTG (Isopropyl β-D-1 thiogalactopyranoside) and grown for 19 h at 37 °C. Cells were centrifuged at 3025 rcf for 10 min. The pellet was resuspended in 5 ml of buffer A: 25 mM potassium phosphate pH 8, 300 mM NaCl, 10% (v/v) glycerol, and 50 mM imidazole. The cells were disrupted by sonication using a Branson 150 sonicator with a 50% amplitude for 10 min using a 15 s pulse, 45 s pause cycle. Cell lysates were centrifuged at 23,700 rcf for 1 h and lysate was injected onto a nickel chelating Sepharose column (Cytiva 6 Fast Flow resin) equilibrated with 2 column volumes buffer A. CASDH was eluted with a linear gradient of increasing imidazole concentration up to 500 mM. Protein was pooled and dialyzed overnight in 25 mM potassium phosphate pH 8, 150 mM NaCl, 10% (v/v) glycerol, and 1 mM DTT (dithiothreitol). BfCASDH was concentrated using an Amicon Ultra 15 concentrator with a 10,000 kDa molecular weight cutoff to a final concentration of 9 mg/ml measured by A_280_ assay (BfCASDH ε = 65,780 M^−1^ cm^−1^; ClCASDH ε = 65,320 M^−1^ cm^−1^) before flash freezing aliquots in liquid nitrogen. This preparation yields 11 mg per L of culture. ClCASDH was purified following the above method except 25 mM Tris pH 8 replaced potassium phosphate in all buffers. The final concentration was 8.5 mg/ml with a yield of 12 mg per L of culture. Size exclusion chromatography was performed by injecting 100 μl of 10 mg/ml (BfCASDH) or 5 mg/ml (ClCASDH) protein on a HiLoad 16/600 Superdex 200 preequilibrated with 25 mM Tris pH 8 buffer.

### Initial rate reactions to determine steady-state kinetic parameters

Initial rates were measured by observing the oxidation of NAD(P)H as an absorbance decrease at 340 nm (ε = 6220 M^−1^ cm^−1^) on an Agilent Cary 60 UV-Vis spectrophotometer. The reaction buffer was 25 mM Tris, pH 7.5 for ClCASDH and 25 mM KPi, pH 7.5 for BfCASDH. Putrescine and DAP were prepared as stock solutions in equimolar Tris HCl buffer and adjusted to pH 7.5. The master mix for varied putrescine and DAP (diaminopropane) contained 1 μM enzyme, and 150 μM NADPH. 2.5 mM ASA (aspartate semi-aldehyde) and varied putrescine and DAP concentrations from 156 μM to 25 mM (BfCASDH) or 9 μM to 40 mM (ClCASDH) were added separately to initiate the reaction and avoid premature interactions between the ASA aldehyde and amines in the reaction mixtures. Master mix for varied ASA contained 1 μM enzyme, 150 μM NADPH, 20 mM putrescine, and varied ASA from 30 μM to 5 mM (BfCASDH) or 39 μM to 2.5 mM (ClCASDH). Master mix for varied NAD(P)H contained 1 μM enzyme and 20 mM putrescine. 2.5 mM ASA and varied NAD(P)H from 0.6 μM to 150 μM were added separately to initiate the reaction. Varied concentrations were made by 2-fold serial dilution and added to the master mix in a cuvette for a total volume of 100 μl. Each experiment was completed as triplicate trials, and the error was calculated as the standard deviation of the parameters measured in each of the trials. Data were fit in Kaleidagraph to the Michaelis-Menten (Equation 1), Hill (Equation 2), or a modified Michaelis-Menten equation (Equation 3) solved in terms of *k*_*cat*_/*K*_*m*_ (19), to determine kinetic parameters.

(1) $$\frac{v_{o}}{\left[E\right]}=\frac{k_{cat}\left[S\right]}{K_{m}+\left[S\right]}$$

(2) $$\frac{v_{o}}{\left[E\right]}=\frac{{k_{cat}\left[S\right]}^{n}}{{K_{m}}^{n}+{\left[S\right]}^{n}}$$

(3) vo[E]=kcat[S]kcatkcatKm+[S]

### Fluorescence binding studies

Data were collected for putrescine or DAP binding by BfCASDH by exciting at 280 nm and measuring emission at 340 nm. All data were collected using an ISS K2 fluorometer. 0.2 μM BfCASDH was mixed with 25 mM Tris pH 7.5 in a quartz cuvette. Ligand was added in a 2-fold series ranging from 156 μM to 80 mM (putrescine) or 156 μM to 300 mM (DAP). The experiment was completed in triplicate, corrected for dilution, and fit in Kaleidagraph using Equation 4.

(4) $$f=\frac{{B_{\max}\left[S\right]}^{n}}{{K_{d}}^{n}+{\left[S\right]}^{n}}$$

### Initial rate reactions to compare homolog chemistries

Initial rates for L-lysine-6-dehydrogenase and saccharopine dehydrogenase activity were measured for Bf- and ClCASDH. Each assay was performed with 1 μM enzyme in reaction buffer, as described above, and in the presence of 500 μM NAD^+^ or NADP^+^ or 150 μM NADH or NADPH. Other substrates were used at a concentration of 1 mM. L-lysine-6-dehydrogenase activity was assayed by combining L-lysine with NAD^+^ or NADP^+^. Saccharopine dehydrogenase activity was assayed by combining L-lysine, α-ketoglutarate, and NADH or NADPH. Each reaction was measured by following the change in dihydronicotinamide absorbance at 340 nm.

### Protein crystallization

All crystals were grown in sitting drop trays composed of a 1 μl: 1 μl ratio of protein and well solution at 4 °C. BfCASDH crystals were grown using 9.5 mg/ml N-terminus hexa-histidine tagged purified protein in a well solution of 0.17 M ammonium acetate, 0.085 M sodium citrate HCl, pH 5.6, 25.5% (w/v) PEG 4000, and 15% (v/v) glycerol with 50 mM ammonium chloride as an additive. Protein was preincubated with 5 mM NADP^+^ and crystals grew anywhere between 4 days and 4 weeks as arrays of thin plates. The well solution was a sufficient cryoprotectant. Crystals were looped and flash-frozen in liquid nitrogen prior to data collection.

### Data collection and structure determination

Diffraction data was collected remotely using Blu-Ice (34) on beamline 9 to 2 at the Stanford Synchrotron Radiation Lightsource. 360° of data was collected at a wavelength of 0.9795 Å with 0.15° oscillations and 0.2 s exposures at a temperature of 100 K. The detector distance was 385 mm. Statistics for data collection and refinement data are listed in Table 2. The data were processed to 1.94 Å in XDS (35). The BfCASDH structure was solved by molecular replacement using Phenix (20) Phaser with 4RL6, chain A as a model. The resulting log-likelihood gain was 1895.88 with a TFZ of 46.0. Initial model building was completed in Phenix Autobuild which placed 778 of 794 residues with R_free_ = 25.32 and R_work_ = 22.44. Density corresponding to NADP^+^ was visible in chain A and B following molecular replacement. Rounds of modeling building and refinement were completed in Coot (36) and Phenix Refine. Waters were placed by Phenix Refine and corrected manually. Once model building was complete, NADP^+^ was added using Phenix Ligandfit. The finished model was refined to R_free_ = 20.87 and R_work_ = 17.86. Ramachandran analysis was performed by MolProbity (37), showing 98% favored conformations with no outliers.

### Structural analysis

Structural comparisons were performed using DALI (38) and with the cealign function in PyMol. The dimeric assembly, interface surface area, and interacting residues were analyzed using PDBePISA (22). The active site pocket was calculated using CASTp (computed atlas of surface topography of proteins) (39). Structure figures were generated in PyMol (PyMol Molecular Graphics System, version 2.1, Schrodinger, LLC). The atomic coordinates and structure factors have been deposited in the Protein Data Bank (accession code 8DEB).

## Data availability

All secondary plots of initial rates may be found in the manuscript or supplementary information. Additional details can be shared upon a request made to the corresponding author. BfCASDH crystal structure data is deposited under the PDB under accession number 8DEB.

## Supporting information

This article contains supporting information.

## Conflict of interest

The authors declare that they have no conflicts of interest with the contents of this article.
