# Supplementary material for: Kinetic and structural characterization of carboxyspermidine dehydrogenase of polyamine biosynthesis
Source: J Biol Chem. 2023 Jul 10;299(8):105033. doi: 10.1016/j.jbc.2023.105033 (PMC10413350; doi:10.1016/j.jbc.2023.105033)
Supplement: Supporting Information [file mmc1.docx]

**Supporting Information**

**Kinetic and structural characterization of carboxyspermidine dehydrogenase of polyamine biosynthesis**

Danielle F. Lee^1^, Nicole Atencio^1^; Shade Bouchey^1^; Madeline R. Shoemaker^1^; Joshua S. Dodd^1^; Meredith Satre^1^; Kenneth A. Miller^1^; and Jeffrey S. McFarlane^1*^

**Table of Contents:**

**S-1** – Figure S1. Purified CASDH PAGE Gel

**S-1 –** Figure S2. CASDH Size Exclusion Chromatograph

**S-2** – Figure S3. BfCASDH Steady-State Kinetic Plots

**S-3** – Figure S4. BfCASDH Fluorescence Binding Plots

**S-4** – Figure S5. ClCASDH Steady-State Kinetic Plots

**S-5** – Scheme S1. Homolog Reaction Schemes

**S-5** – Table S1. CASDH Initial Rates for Alternate Enzyme Activities

**S-6** – Figure S6. BfCASDH Homolog Active Site Comparison

**S-7** – Figure S7. T-Coffee Espresso Structural Alignment

**Figure S1.** **Nickel Affinity-Purified** **CASDH.** ClCASDH M.W. = 44,567 Da. BfCASDH M.W. = 45,077 Da.


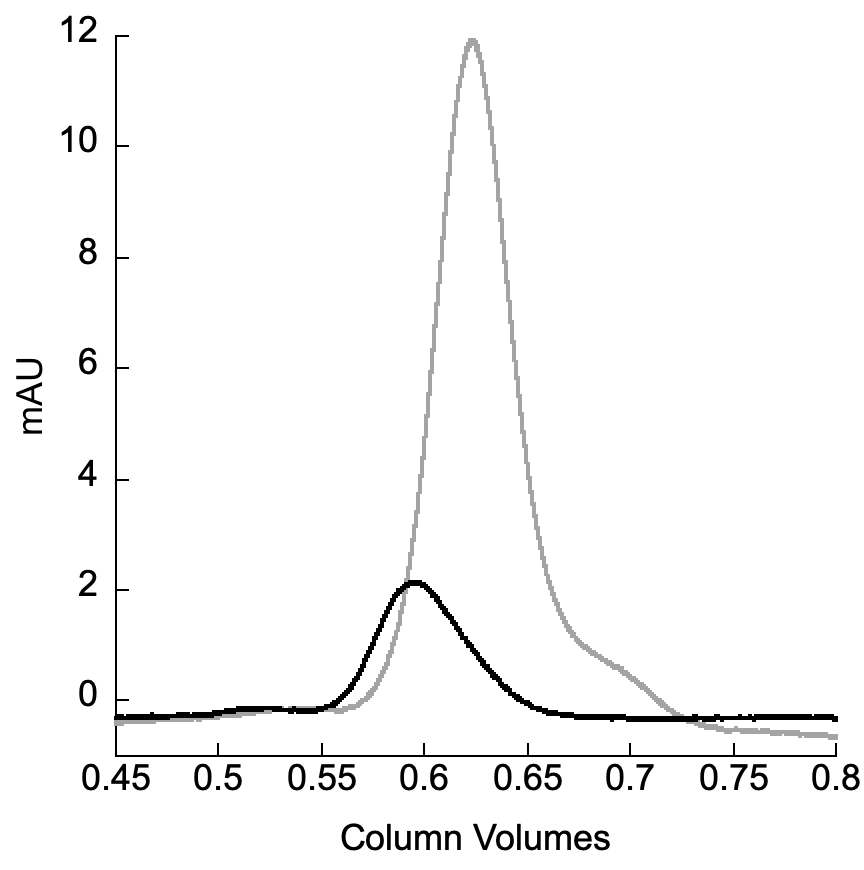


**Figure S2. CASDH Size Exclusion Chromatograph.** Overlay of CASDH elution peaks from a HiLoad 16/600 Superdex 200 pg size exclusion column. BfCASDH (gray) (monomeric M.W. of 45.08 kDa) elutes at 0.62 CV equivalent to a dimeric M.W. of 88.38 kDa. A second shoulder at 0.7 CV is equivalent to the BfCASDH monomer. ClCASDH (black) (monomeric M.W. of 44.57 kDa) elutes at 0.60 CV equivalent to a M.W. of 107.12 kDa.

**Figure S3.** **BfCASDH Steady-State Kinetic Plots.** Initial rates were determined by applying linear fits to the primary plots of [NAD(P)H] (determined using an extinction coefficient of 6220 cm^-1^M^-1^ for NAD(P)H) vs. time). Secondary plots were fit with the Michaelis-Menten equation. Error bars represent the standard deviation of at least three trials. **A)** BfCASDH (1 µM) with 150 µM NADPH, 20 mM putrescine and initiated with aspartate semialdehyde (ASA). **B)** BfCASDH inhibition at concentrations of ASA above 5 mM **C)** Same as for A, but with 2.5 mM ASA and initiated with diaminopropane (DAP). **D)** Same as for A, but with 2.5 mM ASA, 20 mM putrescine and initiated with NADH (inset - adjusted y-axis scale).

**Figure S4.** **BfCASDH Fluorescence Binding Plots.** Polyamine substrate binding by BfCASDH was measured using intrinsic tryptophan fluorescence (Ex. 280 nm; Em. 340 nm). 0.2 µM BfCASDH and varied putrescine (A) or DAP (B) were combined in 25 mM Tris pH 8. Data were collected in triplicate and fit to Eq. 4 using Kaleidagraph. Error bars represent the standard deviation between trials.

**Figure S5.** **ClCASDH Steady-State Kinetic Plots.** Initial rates were determined by applying linear fits to the primary plots of [NAD(P)H] (determined using an extinction coefficient of 6220 cm^-1^M^-1^ for NAD(P)H) vs. time. Secondary plots were fit with the Michaelis-Menten equation. Error bars represent the standard deviation of at least three trials. **A)** ClCASDH (1 µM) with 150 µM NADPH, 20 mM putrescine and initiated with ASA. Sharp inhibition of ClCASDH was observed at concentrations above 2.5 mM. **B)** Same as for A, but with 2.5 mM ASA and initiated with putrescine. **C)** Same as for A, but with 2.5 mM ASA and initiated with diaminopropane. **D)** Same as for A, but with 2.5 mM ASA, 20 mM putrescine and initiated with NADPH. **E)** Same as for A, but with 2.5 mM ASA, 20 mM putrescine and initiated with NADH (inset - adjusted y-axis scale).

**Scheme S1**. Reaction schemes for the enzymes most structurally similar to CASDH.

**Table S1.** CASDH initial rates for alternate enzyme activities (1 µM enzyme).


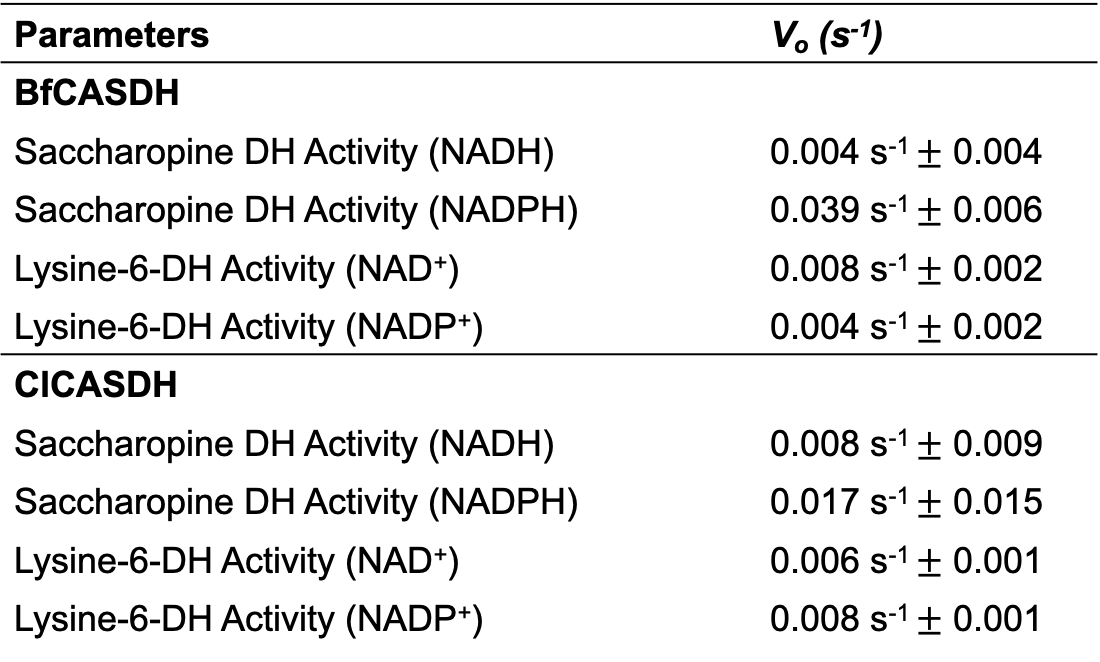


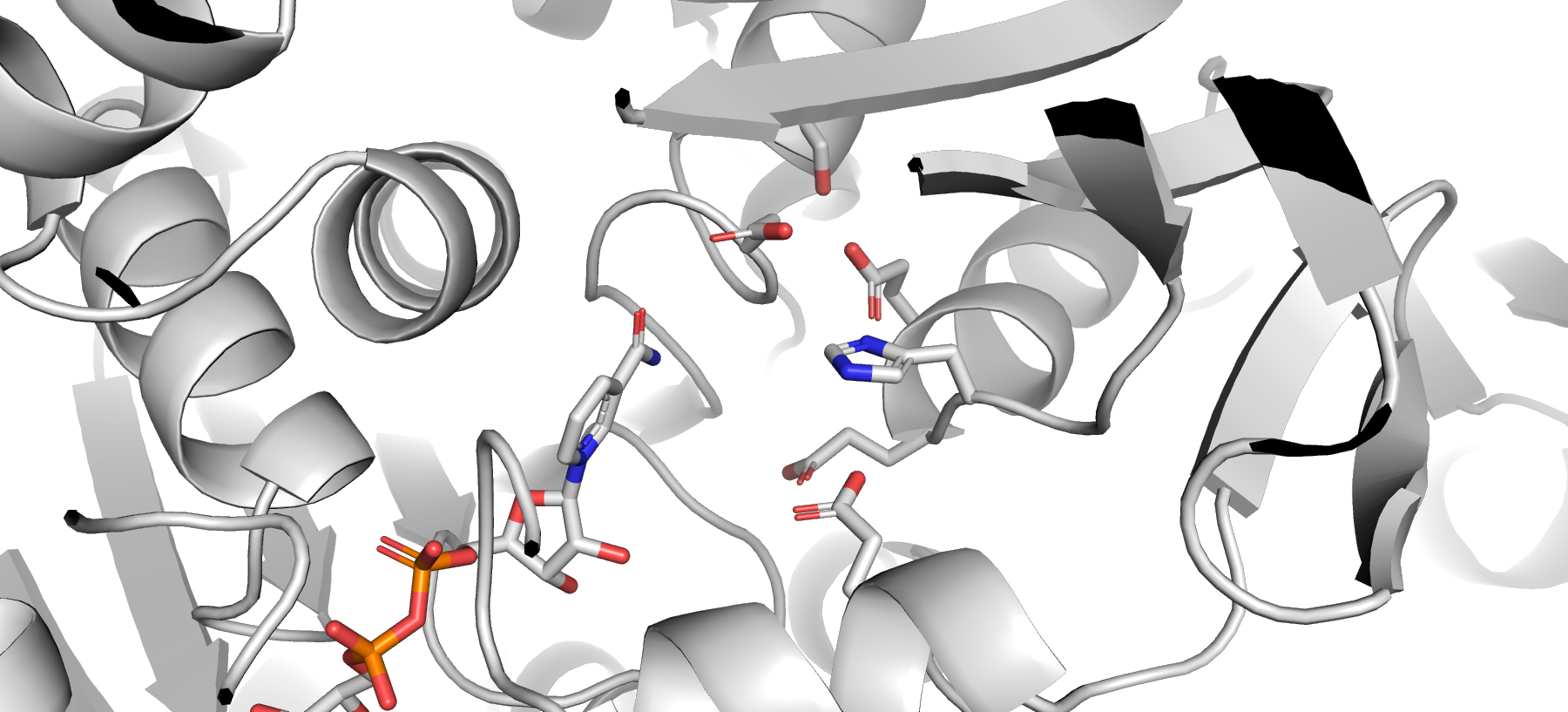

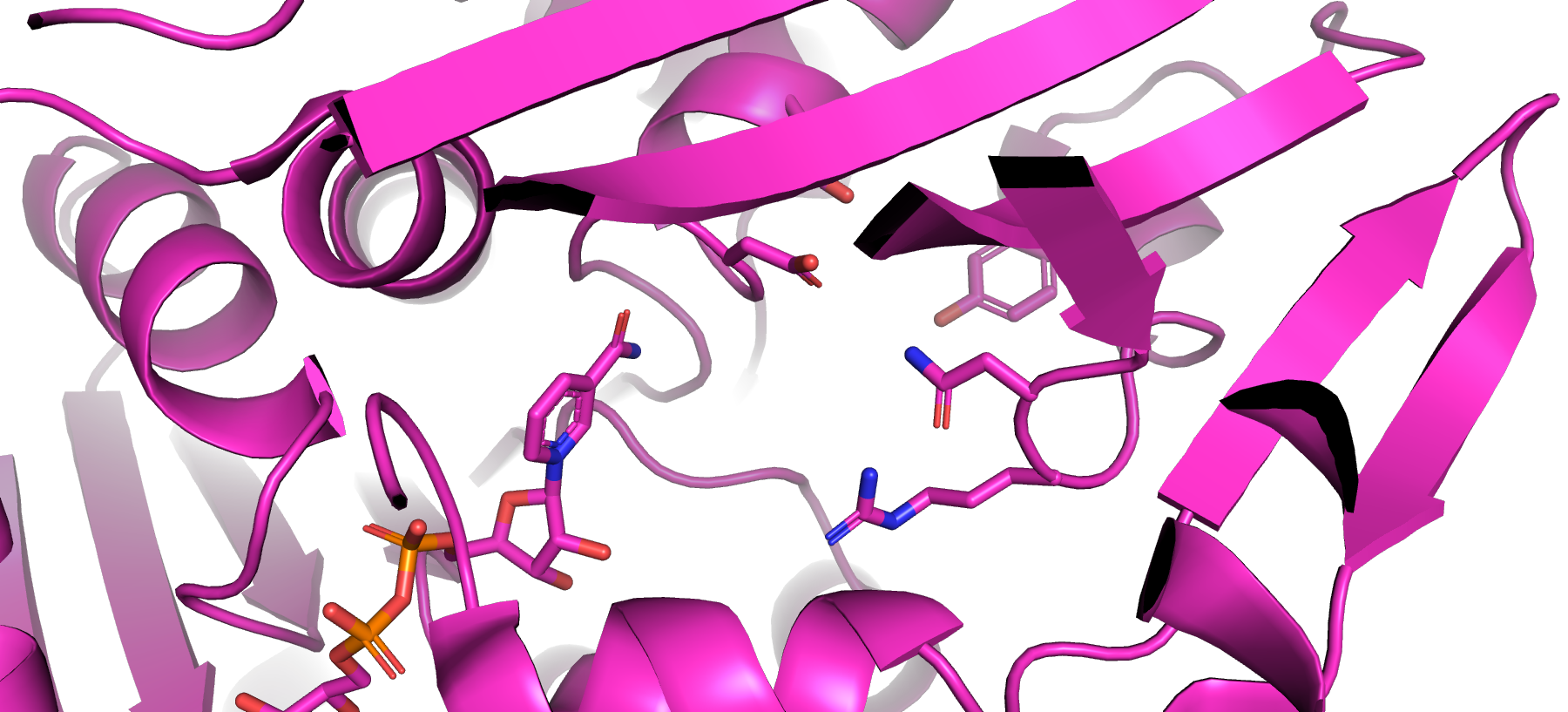

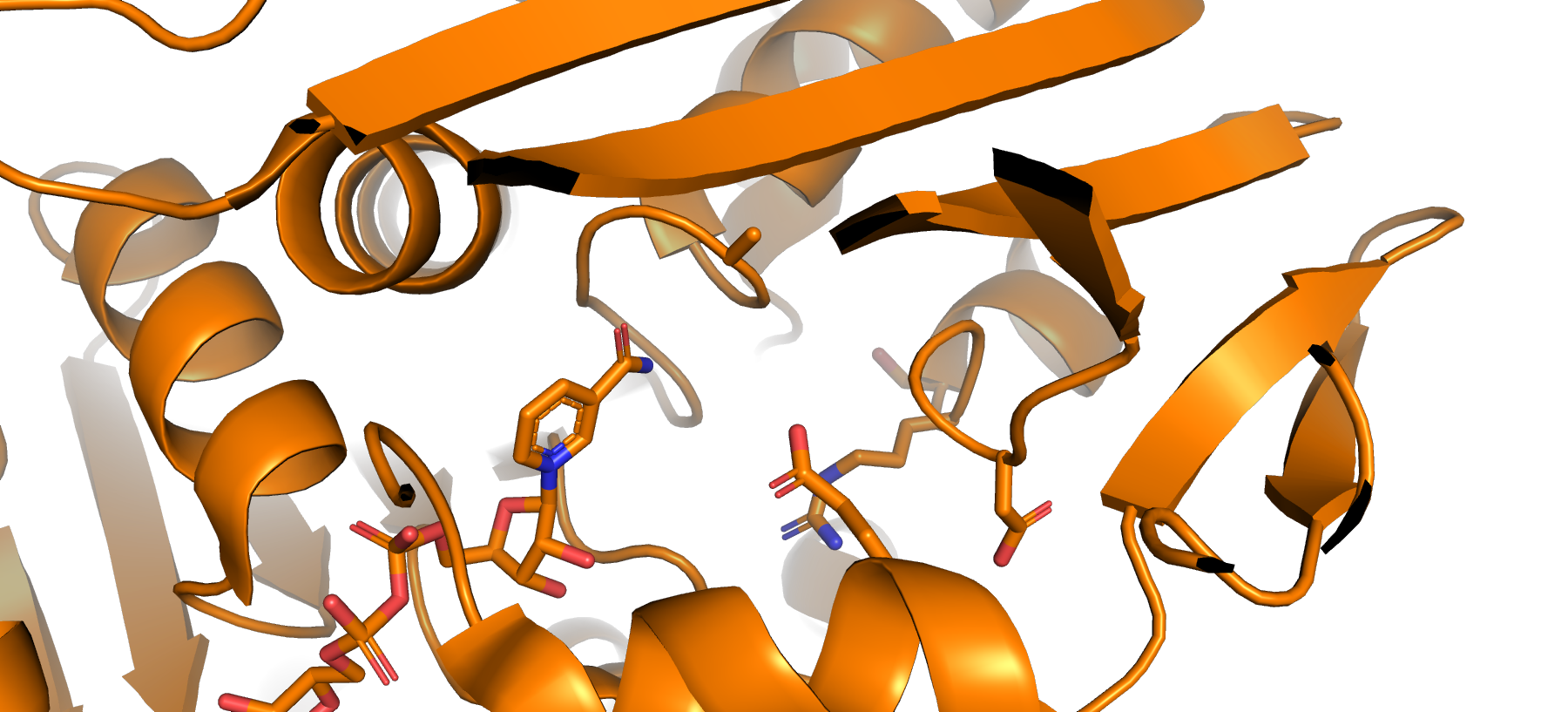

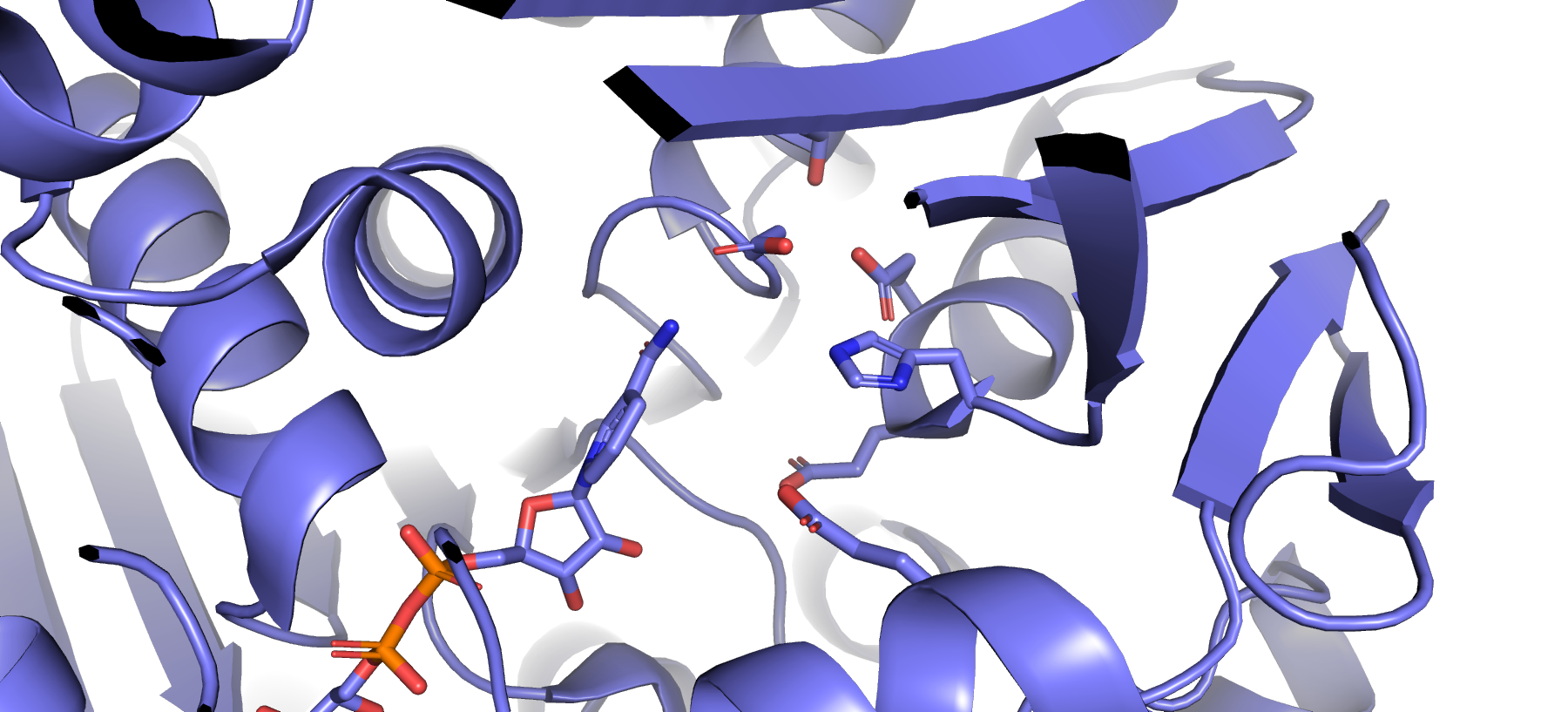

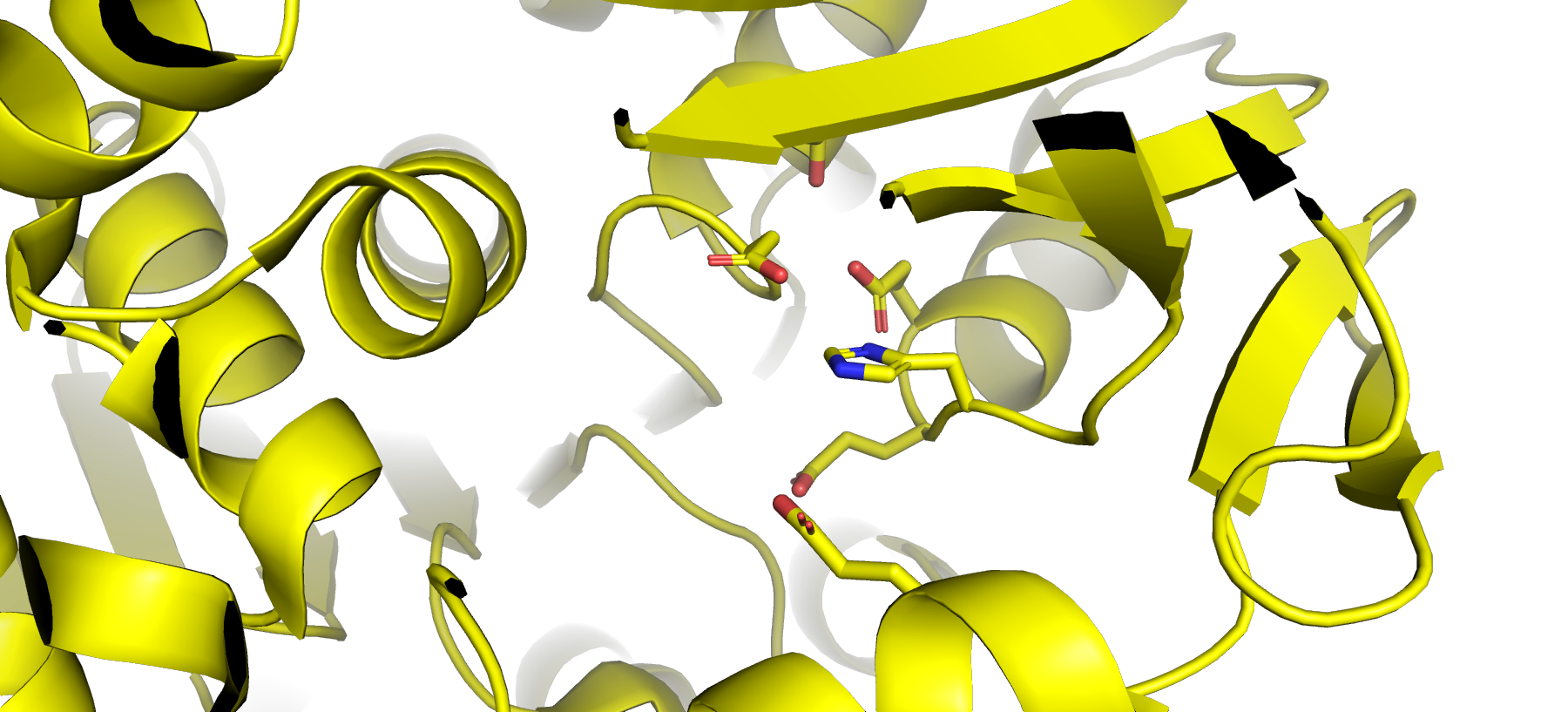


4INA

4RL6

BfCASDH

Lysine-6-dehydrogenase (3ABI)

Saccharopine dehydrogenase (1E5Q)

**Figure S6. BfCASDH Homolog Active Site Comparison**. BfCASDH, 4RL6 and 4INA have very similar active site amino acid arrangements. Saccharopine dehydrogenase (1E5Q) and L-lysine-6 dehydrogenase do not conserve active site residues in the same positions.


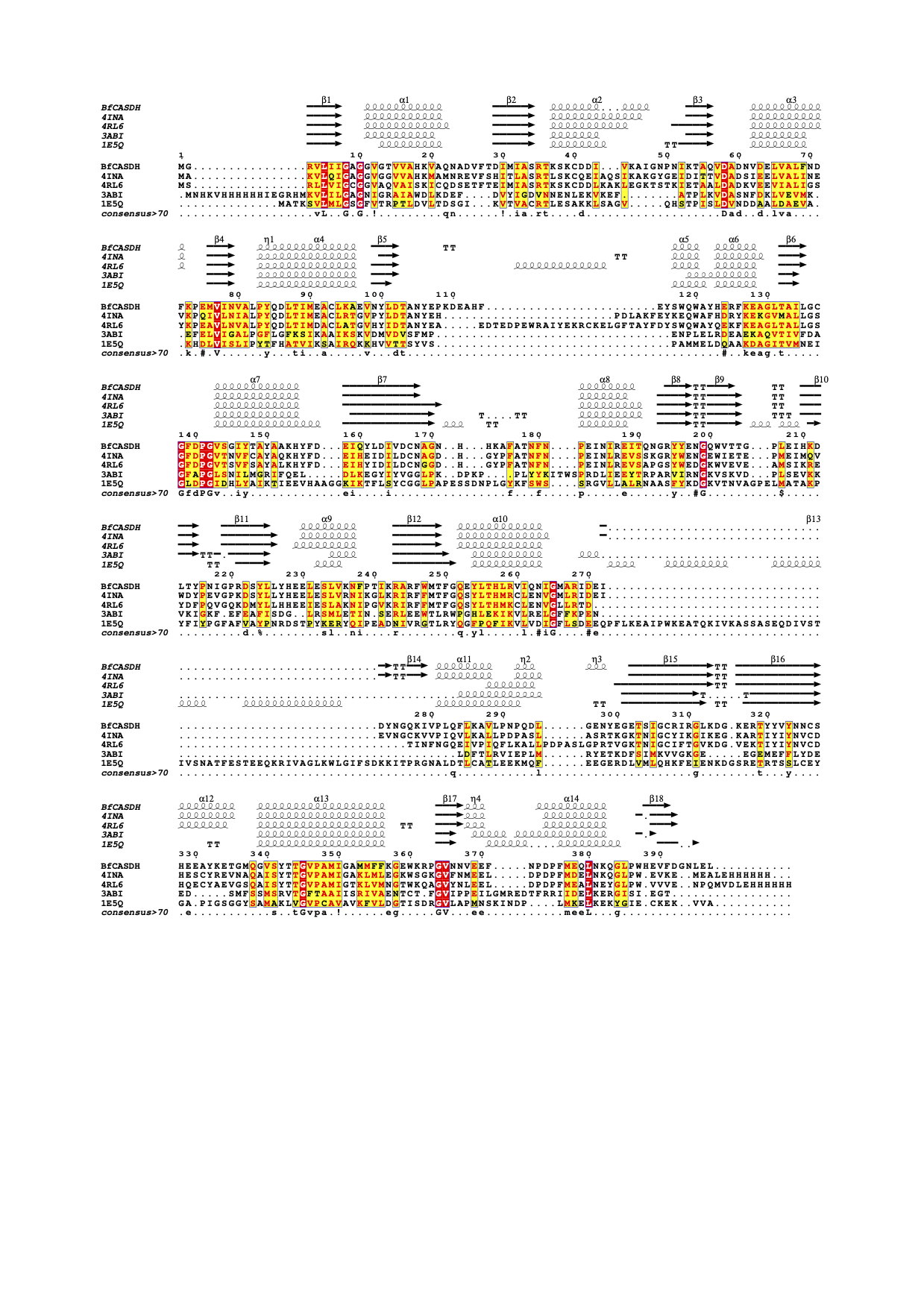


**Figure S7. T-Coffee Espresso Structural Alignment**. Structural alignment of BfCASDH with the four nearest structural homologs in the PDB.
